# Parry-romberg syndrome: about a case

**DOI:** 10.11604/pamj.2017.27.86.12063

**Published:** 2017-06-05

**Authors:** Hanane Oummad, Lalla Ouafae Cherkaoui

**Affiliations:** 1 University of Mohamed V Souissi, Hôpital des Spécialités, Ophtalmology A Department, Morocco; 2 University of Mohamed V Souissi, Hôpital des Spécialités, Ophtalmology A Department, Morocco

**Keywords:** Parry-romberg syndrome, hemifacial atrophy, scleroderma, hyalitis

## Image in medicine

A six-year-old girl presented with skin lesions on the left cheek at 5 years of age. On examination diffuse sclerosis on the left cheek was noted, hypoplasia of left half of the face and deviation of mouth and lips to left side were noted. Investigations show normal blood counts and rheumatoid factor and antinuclear antibody were negative. CT scan of brain was normal. Fundus examination revealed a grade 2 hyalitis. Parry-romberg syndrome (PRS), also known as “progressive facial hemiatrophy” is a rare degenerative condition of the face. It is characterized by progressive but self-limiting unilateral wasting of facial skin, subcutaneous fat, muscle and occasionally bone. It can be associated with various ophthalmic and neurologic complications.

**Figure 1:**
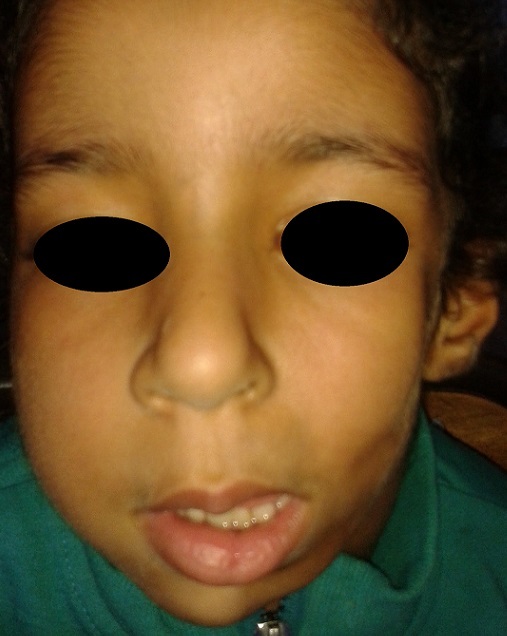
Hypoplasia of left half of the face and deviation of mouth and lips to the left side

